# The perceptions of female breadwinner parents regarding their children’s distance learning during the Covid-19 pandemic

**DOI:** 10.1007/s10639-021-10812-9

**Published:** 2021-11-17

**Authors:** Hibah Khalid Aladsani

**Affiliations:** King Faisal University, Al-Ahsa, Saudi Arabia

**Keywords:** Parent and education, Online learning, Home-schooling, Parental involvement, Low-income families

## Abstract

Covid-19 has affected the everyday educational lives of students, teachers, administrators, and parents. Parents who are living in low-income and disadvantaged communities are probably more likely than others to have been affected by the pandemic in relation to their children’s distance learning. This study focused on the perceptions, predictions, and suggestions of female breadwinner parents from low-income families regarding their children’s distance learning. Data were collected from 12 mothers who participated in a three-stage focus group study. The data from the focus group discussions were thematically analyzed into three categories: (1) financial issues, (2) social and cultural issues, and (3) educational issues. Additionally, the findings presented the breadwinners’ general and technological reasons for their predictions for enhancing education in the future if schools return to face-to-face learning or pursue a blended learning approach. The breadwinners suggested three approaches to teaching and learning for the following academic year. The findings of this study may be useful in the development of educational policies and training programs to provide essential social and technological support to low-income families to address their needs in the online learning environment and to improve digital equity for low-income families who are likely to be educationally disadvantaged.

## Introduction

Covid-19 has changed the educational lives of students, their teachers, and their families. UNESCO estimated that over 90% of students in 200 countries rapidly transitioned from physical schools to online distance learning without access to sports activities or social events (UNESCO, 2020). Students have been forced to become virtual learners as they shelter safely in their homes, and parents, who found themselves working remotely or unemployed, have been compelled to assume new roles as coaches, teachers, and monitors for their children, with no idea how long the situation will continue (Garbe et al., 2020). The rapid transition to online learning has presented several advantages and drawbacks that influenced the physical and mental health of children and their parents (Misirli & Ergulec, 2021).

Prior studies have investigated parents’ experiences, perceptions, and engagement with their children regarding distance learning (Churiyah & Sakdiyyah, 2020; Dong et al., 2020; Garbe et al., 2020; Greenhow et al., 2021; Lee et al., 2021; Misirli & Ergulec, 2021). Most of these studies focused on parents with diverse income levels, educational and socioeconomic backgrounds. However, these drawbacks may be exacerbated, and fewer advantages may be available, for low-income and lower-class families, aggravating society’s digital equity issues (United Nations Children’s Fund, 2020).

This study investigated the perceptions of widowed female breadwinners who were the sole income earners in a low-income household regarding their children’s online learning during the Covid-19 pandemic. The study also explored the breadwinners’ predictions and suggestions for the following academic year. The findings of this study can contribute to improving educational policies and digital equity for low-income families who are likely to be educationally disadvantaged. The paper begins with an outline of the relevant literature and then provides an explanation of the methods and the findings, ending with the limitations, implications and conclusion.

## Literature review

In this literature review, three issues related to the study subject are highlighted. First, female breadwinners and their status in Saudi Arabia are defined. Second, typical online learning is distinguished from distance learning during the Covid-19 pandemic. Finally, parents’ perceptions regarding distance learning during Covid-19 are presented.

### Female breadwinners

A breadwinner is an individual who provides the main financial support for a household, usually a male family member in the traditional model in some western cultures (Chapman, 2004). However, female breadwinners are increasing in industrialized society, challenging traditional western gender norms (Meisenbach, 2010). Meisenbach (2010) studied the experiences of female breadwinners and gendered identity in family spaces, finding that the research participants felt a sense of control and power on the decision making in their lives. The participants also experienced pressure as the prime breadwinner in their family and worried about what could happen to their families if they lost their jobs. Some participants felt guilt and resentment regarding their dereliction of house duties.

In Saudi tradition, the context of this study, males should be the breadwinner for the family. However, the entry of women into the labor market has changed this dynamic. The Saudi General Authority for Statistics (2020) stated that the percentage of women in the total workforce in 2020 was 35%, compared to 65% males. Several factors encourage Saudi women to participate in financially supporting their household. Several factors encourage Saudi women to participate in financially supporting their household. These factors include an increase in living expenses, improved family well-being, heightened self-esteem, and coping with the financial crises that low- and middle-income families often face (Samargandi et al., 2019).

### The difference between typical online learning and online distance learning during Covid-19

Successful online learning requires careful instructional planning and design, quality teachers trained in online instruction, and high-level technology resources (Hodges et al., 2020) and, thus, necessitates plenty of time to prepare, ranging from months to years (Daniel, 2020). Typical online learning provides institutions, teachers, students, and parents with the opportunity and the time to prepare for and adapt to this type of learning (Daniel, 2020). However, the need to implement distance learning during Covid -19 presented a unique situation, as schools and universities around the world were forced to fully transition to an online learning format quickly without the essential preparation (Al Lily et al., 2020; Daniel, 2020; Hodges et al., 2020).

### Parents’ perceptions regarding online distance learning during Covid-19

The following sections address the advantages for and drawbacks of online learning during Covid -19 from parents’ perspectives as presented in the literature.

#### The advantages of distance learning during Covid-19

There are several advantages discussed by parents in prior studies. For instance, distance learning during the Covid-19 pandemic, maintained children’s learning while safely sheltering them at home (Bubb & Jones, 2020; Garbe et al., 2020; O'Keefe et al., 2020). Another opportunity is that distance learning requires limited movement, consequently, offers learning advantages to those who cannot attend traditional schools, such as disabled students or those who need to travel frequently (Al Lily et al., 2021). 

The transition to online distance learning during the pandemic has expedited the adoption of technology in K-12 schools and has accelerated young students’ acquisition of digital skills in a short period of time (Bubb & Jones, 2020; Ewing & Cooper, 2021; Misirli & Ergulec, 2021). Bubb and Jones (2020) found that distance learning improved parents’ digital skills such as using digital devices, communicating with others, and searching for information. The researchers attributed the improvement to the online training workshops and support provided by schools in the use of digital resources (Bubb & Jones, 2020).

Another advantage of distance learning mentioned by Bubb and Jones’s (2020) was improved self-regulation among children with respect to their learning. In their study, parents reported that their children had become more independent after taking responsibility for their own learning without constant interventions from teachers. However, the findings of several other studies (Dong et al., 2020; Garbe et al., 2020; Misirli & Ergulec, 2021) conflict with this observation, reporting instead insufficient self-regulation in children in their distance learning, which consequently imposes a further burden on parents in supporting their children’s education.

One of the benefits of distance learning is that it allows parents to more effectively assist in the development of their children’s educational process, as parents can see firsthand what is happening inside the classroom, how teachers teach and communicate with their children, and, importantly, how their children learn and behave classes (Bubb & Jones, 2020).

Distance learning can also strengthen the relationship between parents and school staff via the use of social networking sites, such as WhatsApp and Facebook, to communicate about children’s assignments and the academic process in general (Bhamani et al., 2020).

#### The drawbacks of distance learning during Covid-19

The sudden shift to distance learning has made many parents feel overwrought and unready (Churiyah & Sakdiyyah, 2020; Dong et al., 2020; Garbe et al., 2020). Parents have faced many difficulties in caring for their children while at the same time attempting to support their education, all the while working remotely from home (Bhamani et al., 2020; Garbe et al., 2020).

Parents are mainly responsible for supporting their children’s engagement in distance learning because they are the only ones who can physically sit with their children during the pandemic (Misirli & Ergulec, 2021). Overwhelmingly, mothers are responsible for the care and home-schooling of their children (Al Lily et al., 2021). 

Adding these new responsibilities to existing ones creates competition over limited time and energy, with parents struggling to balance all of these responsibilities while ensuring adequate support for their children’s distance learning (Dong et al., 2020; Garbe et al., 2020; Karakose et al., 2021). In addition, as most parents have neither studied nor trained to be educators, they suffer from a shortage of academic knowledge about the content of their children’s curricula and pedagogical basis, which greatly impedes their capacity to support their children’s learning (Dong et al., 2020; Garbe et al., 2020; Sahin & Shelley, 2020).

Lack of self-regulation and internal motivation on the part of many children imposes immense pressure on parents to motivate them and manage their time and space (Dong et al., 2020; Garbe et al., 2020). Children’s lack of internal motivation relative to online learning during the Covid-19 pandemic can be attributed to various factors. For example, technical issues related to maintaining an Internet connection led children to lose their motivation to learn (Dong et al., 2020). Further, distractions at home and the decrease in social interactions with peers and teachers in online learning settings impacted students’ enthusiasm for learning (Garbe et al., 2020). Thus, parents have found themselves doing more monitoring, motivating, and engaging in their children’s distance learning, which can cause conflict and damage their relationship with their children (Misirli & Ergulec, 2021).

Distance learning increased families’ financial spending, as money had to be allocated toward purchasing the technology necessary for children to participate in distance learning, including Internet service and computer devices, such as laptops, tablets, or mobile phones (Abuhammad, 2020). Purchasing a computer device for each child may pose an increased financial burden to families with multiple children (Al Lily et al., 2020). Restricted financial resources and lack of sufficient internet capacity impose an even greater burden on parents in supporting their children’s distance learning (Garbe et al., 2020). Gandolfi et al. (2021) found that Covid-19 and distance learning have broadened the digital divide in the educational system for students from low-income families.

## The purpose of the study and research questions

Covid-19 has affected all aspects of life such as the economy, health and education across the globe (Karakose et al., 2021). Parents who are living in low-income and disadvantaged communities likely have been affected by the pandemic more than others in relation to their children’s online learning (Greenhow et al., 2021). Families living under low socioeconomic conditions may not be able to benefit from the opportunities provided by educational technology and distance learning tools, leading to a significant interruption in education (Sahin & Shelley, 2020). Therefore, to provide essential social and technological support to disadvantaged families, studies have been conducted to better understand parents’ perceptions regarding their children’s online learning (Gandolfi et al., 2021; Greenhow et al., 2021; Sahin & Shelley, 2020). However, none of these studies focused on low-income families who depend on a single parent. Thus, this study investigated the perceptions of female breadwinners from low-income families regarding their children’s distance learning.

The following research questions guided this study:

- Q1: What are female breadwinners’ perceptions of their children’s distance learning during the Covid-19 pandemic?
- Q2: What are female breadwinners’ predictions about and suggestions for the educational system in the subsequent academic year?

## Methodology

This study applied a qualitative design to gather particularly detailed data. Focus groups were the method of data collection.

### The context of the study

King Faisal University (KFU) in Al-Ahsa in Saudi Arabia has been offering a training program entitled “Initiative of Empowering the Breadwinner Mother” in cooperation with the Al-Bir association, every year specifically for widows with children in low-income families who receive no financial support (Alshuaibi, 2021). Al-Bir association provides financial and moral support to widows with children in low-income families and encourages breadwinner mothers to search for jobs or become involved in productive families to secure decent lives for themselves and their children (Takamol Center for Orphans Care, 2021).

This program has sought to empower these mothers by making a diploma training program available to assist them in preparing to pursue better careers, to increase their earnings, and to become self-reliant. The program was also designed to enhance the scientific competence of breadwinner mothers, enabling them to address the problems they may face in raising their children and contribute to the creation of a suitable educational and scientific environment for the upbringing of these fatherless youth (Takamol Center for Orphans Care, 2021).

The researcher taught one of this program's courses titled “Computer and Mobile Applications,” taking advantage of the opportunity to teach a more unreachable population to investigate their perceptions of, predictions for, and suggestions regarding their children’s distance learning. She intended for her research findings to make the voices of this marginalized population heard by people who are in positions of authority with responsibilities related to human equity on issues concerning this population’s rights and circumstances in relation to their children's distance learning.

### Participants

The number of trainee mothers reached 15; however, three mothers were absent on the day of data collection. Thus, 12 mothers participated in this study. The focus group approach was carried out face to face, at the university campus with the adhering to Covid‐19 precautionary measures.

Table 1 shows the background characteristics of the participants. More than half of the participants (*n* = 7) had only a high school degree.

**Table 1:** Background of participants (*n* = 12)

Academic degree	N
Bachelor’s degree: - Islamic Studies - History - Home Economics	5 1 2 2
High school degree	7

Table 2 shows the study level of the participants’ children stratified by gender. As shown in the table, primary and secondary school children constituted the majority of the breadwinners’ children: 24 children out of 34, or 70% of the total number of children.

**Table 2:** Study level of participants’ children by gender

Children’s study level/ gender	Primary	Secondary	High school	University	Total number of children
Girls	11	5	4	3	23
Boys	3	5	3	0	11
Total	14	10	7	3	34

### Data collection method

Data were collected using the face-to-face focus group method, which involves interviews with more than one interviewee (Bryman, 2016). Focus groups were selected as the data collection method as they generate valuable discussions and interactions between group members (Gray, 2014). The participants in this study shared similar life circumstances and experiences as breadwinner mothers who cared for children during their distance learning. Unlike individual interviews, focus groups allow for the collaborative generation of data, permitting participants to add to and comment on the views expressed by others (Gray, 2014). In the individual interview, the participant is asked about the reason for presenting his or her opinion, while in the focus groups, participants are offered the chance to examine and explain each other’s reasons for presenting a specific opinion (Bryman, 2016). (This practice emerged several times in the data, as mothers commented on or explained other mothers’ views.)

### Data collection procedure

**Fig. 1:**
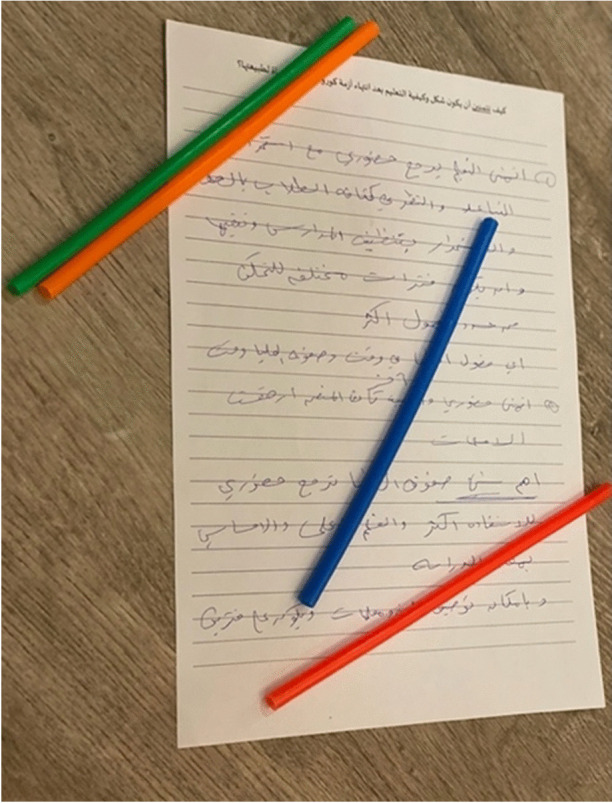
Each group was provided with four different colored straws

The researcher divided the participants into three groups. Each group had four mothers who were provided with colored straws red, orange, green, and blue (see Fig. 1).

The focus groups were conducted in three stages. During the first stage of the research, each mother had the opportunity to think individually consider the provided questions, which encouraged her later to effectively participate in the group discussion. The researcher applied this idea as a solution for one of the common challenges in the focus group method, which is getting equal participation from each group member (Bryman, 2016).

Second, each group was asked to discuss their perceptions regarding their experience of their children’s distance learning during the Covid-19 pandemic, as well as their predictions and suggestions about the following academic year. In this stage, the researcher was involved with each group, listening, writing down the responses, and posing further questions.

Third, the groups were rearranged based on the colored straw each mother received. Accordingly, there was a red group, an orange group, a green group, and a blue group, each comprising three mothers. The mothers were instructed to share their views again regarding the provided questions. Rearranging and mixing groups helped the participants listen to others’ views and then modify their own views or simply acknowledge their agreement or disagreement with a point they would not have considered without the opportunity to hear from others. Thus, this method elicited a variety of opinions related to participants’ children’s distance learning (Bryman, 2016).

The researcher wrote down the resulting data immediately as the mothers were uncomfortable having their voices recorded. Further, to ensure the participants’ anonymity, all of their responses were written down anonymously without any mention of their names. The mothers were informed that they had the right to decline to answer any question and that they could withdraw from the research at any time without any consequence. Unrecording and subsequently non-transcribing the data for the focus groups may be a limitation of this study, as the researcher had difficulty taking notes and managing the discussion concurrently (Bryman, 2016). However, mixing and rearranging groups in the third stage assisted with recovering some missing data.

The three-stage focus group approach encouraged the mothers to speak about and discuss their views in a free, enjoyable, and friendly environment. The three stages also generated intensified, synergistic, and cumulative data due to the repeated discussions between the mothers. The discussions lasted for 3.5 h, from 8:00 a.m. to 11:30 a.m., which is the standard class period in this course. Thus, the focus group method permitted the collection of rich and in-depth data in a brief amount of time (Creswell & Poth, 2018).

### Data analysis

The research data were manually analyzed with consideration of the research questions. The data were coded thematically to support each research question using an inductive approach to detect themes and patterns in the data (Braun & Clarke, 2006). The researcher followed the six steps of the thematic analysis approach provided by Braun and Clarke (2006), starting with reading the data several times to familiarize herself with the data and then generating the initial codes. The researcher summarized the basic topic of a passage in a short phrase name, defined by Saldaña (2016, p. 102) as “descriptive coding.” The third step involved collating codes into potential themes and then reviewing these themes to ensure that each was relevant to the coded extracts. Next, the themes were organized and refined to fit the research objective and questions. Finally, the report on the data analysis presented in the findings section was produced (Braun & Clarke, 2006).

### Ensuring trustworthiness

The term “trustworthiness” is used instead of positivist theoretical perspectives of validity and reliability (Lincoln & Guba, 1985). To ensure the trustworthiness of the study, the researcher followed various strategies provided by Lincoln and Guba (1985). For example, in the lecture that followed the focus group session, the researcher provided the participants with a summary of their focus group discussions for their review to determine the accuracy of the records, called member checking (Lincoln & Guba, 1985). Additionally, in-depth descriptions of the data collection and analysis methodologies are provided to show how the data ultimately led to the construction of the findings (Lincoln & Guba, 1985).

## Findings

The findings from the data were organized based on their relation to the research questions.

### Themes related to the first research question

- Q1: What are the breadwinners’ perceptions of their children’s distance learning during the Covid-19 pandemic?

The mothers’ perceptions of their children’s distance learning during the Covid-19 pandemic fell under three themes: (1) financial issues, (2) social and cultural issues, and (3) educational issues. The subthemes for each and the kind of perceptions classified under each subtheme are illustrated in Table 3.

**Table 3:** Themes and subthemes for breadwinners’ perceptions of their children’s distance learning

Themes	Subthemes	Type of Perception
Financial Issues	• Educational expenses of the Ministry of Education • Family financial issues	• Positive • Mixed
Social & Cultural Issues	• Extra work for mothers • Health issues • Privacy issues • A solution for some social dilemmas	• Mixed • Mixed • Negative • Positive
Educational Issues	• The quality of education • Primary and secondary students • Educational benefits	• Negative • Negative • Positive

#### Financial issues

Participating widowed mothers presented their perceptions about distance learning in relation to financial issues during the focus group sessions. This theme involves two subthemes: educational expenses for the Ministry of Education and family financial issues.

##### Educational expenses for the ministry of education

The mothers discussed the benefit of the reduction of educational expenses for the Ministry of Education. As one mother said: “The Ministry of Education profits economically from the distance learning. It does not pay for the buses or drivers because no students are coming to the schools. The electricity bills are low due to the absence of the students.”

##### Family financial issues

As breadwinners for their families, all of the mothers were anxious about financial issues. They were grateful to the charity organization Al-Bir association for providing each of their children with a new laptop. However, one mother said: “These devices are poor quality. They freeze and damage so quickly. So, I bought other devices for my children from a high-quality brand, which badly affected my budget.”

Mothers worried about the frequent damage of computer hardware, such as speakers, microphones, and keyboards. The mothers discussed how these technical issues negatively affected their children’s learning, at times causing them to miss lessons.

In addition, the mothers appreciated the reduction of in-school expenses, but they were also concerned about other expenses incurred by distance learning. As one mother explained:

We do not need to buy school uniforms, bags, stationery, meals, and driver wages for distance learning. However, we spend much money on internet service. I was forced to update my home internet package to meet my children’s educational requirements.

Regarding internet service, the mothers complained about the poor connection of their service, which distracted their children from learning. Some mothers explained that their children were marked as absent due to a failed or poor internet connection. They discussed how their children were saddened when their teacher could not hear them and marked them as absent.

Another financial issue that concerned breadwinner mothers was the increase in electricity fees at home. One mother explained this increase:

Distance learning makes a mess of our lives. Each child wants a separate room to focus on his/her study and not be distracted by siblings. It is hard to allocate a specific room for each child due to the small size of our home. Moreover, the electricity bill has increased by double. All of the lights and air conditioners are turned on in each room in the house.

Another mother commented: “Adding to that, our children stay at home all day due to the lockdown and concerns over the virus, which means more use of electricity.”

#### Social and cultural issues

Widowed mothers discussed several issues related to social and cultural aspects of their children’s distance learning. This theme was divided into four subthemes: extra work for mothers, health issues, privacy issues, and a solution for some social dilemmas.

##### Extra work for mothers

Distance learning during the Covid-19 pandemic has transferred the place of learning from the school to the home. Learning at home has been accompanied by extra work for mothers besides their ordinary housework and job demands. Therefore, the mothers felt overwhelmed. Some mothers explained that they had evening jobs that started at the same time as the beginning of their children’s educational platform, which is 3:00 p.m. in Saudi Arabia. They suffered from having to work during their children’s school time, as explained by one mother:

I must stay with my son during his attendance of online classes, otherwise he will leave the educational platform and play. But my part-time job requires evening work on some days. I must leave my young children with their older siblings, who honestly cannot manage this situation as I can.

Another mother explained the reason for monitoring her children during their online learning: “Children cannot focus for a long time on digital screens. I am tired of chasing them every minute when they play instead or simply minimize the platform page and open another page to watch YouTube videos.”

On the other hand, some mothers found some benefits of staying beside their children while they studied even though it brought extra work for them. As one mother explained: “I like to stay with my daughters during their learning on the platform. I want to see and evaluate how they learn, listen, and interact with teachers. I want to see their academic performance inside the classes,” Another mother added: “So, I can give my children the help they need.” Yet another mother commented: “It is great to see the teaching style of the teachers and how they behave with my kids.”

##### Health issues

Distance learning from home can protect children from healthy dangers outside the home. All of the mothers believed that transitioning from face-to-face classroom learning to distance learning can help prevent the spread of Covid-19.

Distance learning can also protect students who have underlying health conditions. As one mother said:

My daughter has diabetes. She needs to take her medication regularly. I used to call her teacher’s mobile phone or visited the school to do that. Once, her teacher forgot that and my daughter went into a diabetic coma. It is easier to give her the care she needs with distance learning at home.

Eating healthy food and using clean bathrooms at home were other examples mentioned by mothers as ways to protect their children from outside dangers. Some mothers mentioned not mixing with bad people as a benefit of distance learning in that it safeguarded their children from moral danger.

On the other hand, negative health issues were reported by the mothers. Several mothers noted that their children were becoming visually impaired due to spending too much time viewing digital screens. Other mothers discussed the pain their children suffered in their necks, shoulders, and backs from having to bend over digital devices for so many hours. Some mothers reported that their children had become addicted to digital devices, wanting to use them all the time even after the educational platform had ended. About this, one mother said: “My son increasingly clung to his mobile phone this year. If I take it from him, he gets angry.”

##### Privacy issues

Distance learning raised privacy issues for those living at home. For instance, turning on the microphone to communicate and using the camera for face-to-face interactions exposes home affairs. About this, one mother said: “Turning on the camera makes me scared of accidentally appearing to others who are in my son’s class.” Another mother commented: “Oh, all of the home secrets became public because of this distance learning.” Amused, another mother stated: “We had to talk in whispers inside our own home for fear of an opened microphone or camera.” One mother asked: “Why do you stay in the same room as your kids during their study?”. To this question, another mother answered: “If I leave, my child will leave the platform too. I must mentor him.” Another mother added: “There is no empty room to stay alone. Even my own bedroom is occupied by my children.”

##### A solution for some social dilemmas

Some mothers appreciated a benefit of distance learning to decrease the car and bus accidents as well as a decrease in traffic jams, especially in the morning and noon rush hours. About this, one mother stated: “We no longer hear about school bus accidents.” Another mother explained: “The reduction of these accidents is due to the absence of morning travel to schools every day and the crazy driving by the delayed people to catch the school bell.”

Moreover, distance learning provides a solution to school closures for emergency situations, such as bad weather or pandemics. As one mother claimed: “Usually, schools are closed during bad weather, such as dust storms or heavy rain. This year, schools never closed due to bad weather because of the distance learning at homes.”

Distance learning has also reduced some bad social behaviors in the school communities. One mother remarked: “Harassment and bullying have been limited in distance learning.” Another mother commented: “Although there is cyberbullying, it is nothing compared to bullying in face-to-face learning.”

#### Educational Issues

Widowed mothers’ perceptions about their children’s distance learning in relation to educational issues were also addressed during the focus groups. Educational issues were categorized according to three subthemes: the quality of education, primary and secondary students, and educational benefits.

##### The quality of education

The mothers discussed the differences between distance learning and face-to-face learning for their children. The main difference they mentioned was the focus of studying and the quality of education. The mothers demonstrated that their children had been more focused in face-to-face classes and thereby gained considerably more understandable knowledge than in online classes. The mothers also noted several distractions in online learning, such as sleeping, eating, continuously going to bathroom without need, browsing websites, or simply running away and playing outside.

The mothers expressed intense concern regarding the quality of education in distance learning and the extent to which their children could comprehend their lessons. A few mothers attempted to address this issue by reviewing lessons with their children to ensure they did not miss anything or had not misunderstood their online lessons, which led to extra work for these mothers, as discussed earlier. Most of the mothers declared their insufficiency in providing the necessary educational assistance to their children due to the lack of a university degree or the complexity of the children’s curricula.

The mothers discussed the reasons why their children were more distracted in online learning. First, the lack of self-discipline. As one mother said: “What do you think when kids have digital devices and internet in their hands? Absolutely, they use them for entertainment, not studying.” Another mother stated: “Every time I look at my son’s device, I see social network sites, and I hear a weak voice of the teacher in the background.” Other mothers believed that online learning was hard for young children who could not understand what was good for them, got bored quickly, and could not sit for many hours in front of digital screens, especially as they were missing physical and social connections with their peers and teachers.

Another reason was the negative changes that occurred in the relationship between students and their teacher in distance learning. As one mother said: “There is no prestige of or fear from teachers in distance learning as in face-to-face learning.” Another mother added: “The communication between teachers and students is lost in this distance learning. The young students are noisy and messy. The teacher cannot control them.”

Such bad management was due to poor preparations on the part of teachers for distance education. As one mother claimed: “In the beginning of the distance education, many teachers found difficulty using the online platform, which caused messy online classes and was confusing for students.” Another mother explained: “Unfortunately, many teachers do not have the required digital skills. They cannot deal with computer skills or online teaching.”

The third reason was absence of physical social communication between the children and teachers or peers. About this, one mother argued: “There is no connections with peers, no making new friends, no social activities, our kids feel bored.”

The absence of physical connection has led to another problem as reported by mothers: the misjudgment of students by their teachers. As one mother said: “My daughter is distracted by her peers’ noise in the online classroom. Her teacher could not hear her voice, so she deducted from her marks.” Many mothers mentioned this problem as being unfair. As one mother explained: “Some teachers cannot manage online classrooms. My son cried one day because he could not complete his answer and the teacher gave him a zero. My son complained that selfish students always interrupt anyone who tries to answer.”

An additional reason that affected the quality of education was cheating. Cheating occurred in both assignments and exams. One mother claimed: “There was no respect or preparation for online exams as in face-to-face exams. No one studies for exams because all students open their books in either open book or close book exams.” Cheating resources were not limited to books alone. Websites, friends, big brothers and sisters, and even mothers often assisted in the cheating process. Most of the mothers declared that they had helped their children in completing assignments and conducting exams. One mother defended her behavior: “My poor daughter usually feels unconfident. So, when her teacher asks her, she is horrified and looks at me with tears. Consequently, I tell her the answers.” Other mothers explained that distance learning was not worth it, and that no good knowledge had been gained. These mothers thought their children had to get high marks at least in the current year, which is why they helped their children with their assignments and exams. One mother, however, did not agree with this justification. She said: “You will make your children lazy and unresponsible. You also put additional efforts into your already busy daily schedule.”

##### Primary and secondary students

The mothers explained that their young children, especially primary and secondary students, were suffering the most from distance learning, whereas those in high school or university worked well in the distance learning format. Some mothers who had children in year one raised additional concerns regarding their children’s literacy skills. About this, one mother said: “The academic year has ended and my poor child is still unable to write well.” Another mother advised her: “You have to wait until next year, hopefully to start face-to-face learning, not online learning, as I have done for my little one.”

##### Educational benefits

Despite all of these negative effects of distance learning on students, the mothers mentioned several benefits their children had gained from online learning. Heightened digital skills was the benefit mentioned most often by the mothers, who noted how much their children had improved in using computers and other digital devices. One mother said: “My children have become creative in using computers. In the second month of online learning, they started to create private rooms on Microsoft Teams to communicate with their friends.”

Some mothers appreciated the variety of assessments with which their children were evaluated, which allowed them to collect marks more reliably than with exams alone.

Distance learning also benefited students who had movement disabilities. These students could attend classes without having to commute to and from school.

The mothers mentioned that their children felt more confident in online learning than in face-to-face learning. As one mother said: “My children pray to be in online learning next year. I pray against what they said. They are happy to stay at home, eating, sleeping, and playing during online classes; otherwise, these activities are forbidden in face-to-face learning.” Almost all of the mothers shared the desire for their children to return to face-to-face learning the following year.

### Themes related to the second research question

Q2: What are female breadwinners’ predictions about and suggestions for the educational system in the subsequent academic year?

The findings related to the second research question fell under two themes: (1) predictions and (2) suggestions. The mothers believed that education would be enhanced in the future if schools returned to face-to-face learning or pursued a blended learning approach. They presented several reasons, each of which represented one of two types of reasons: general or technological. The mothers suggested three approaches to education for the following academic year: offering students’ family a choice between distance or face-to-face learning; dividing school time into two periods; and applying blended learning, depending on the school’s curriculum, student ages, and/or selected days.

The subthemes for each theme are illustrated in Table 4.

**Table 4:** Themes and subthemes of breadwinners’ predictions about and suggestions for the educational system for the subsequent academic year

Themes	Subthemes
Breadwinners’ Predictions About Enhancing Education	Reasons for breadwinners’ predictions: • General reasons: 1- Reclaim education for its prestige 2- Increase level of comprehension due to physical attendance 3- Fair marks for students 4- Free time for mothers to rest and get energy to assist with their children’s learning • Technological reasons: 1- Increase digital skills of children and their families 2- Increase teachers’ digital skills 3- Increase use of Internet at schools 4- Maximize benefits of Ministry of Education educational platform 5- Increase value of the computer curriculum and digital skills
Breadwinners’ Suggestions for Subsequent Academic Year	• Offer a choice between distance and face-to-face learning • Divide school time into two periods • Apply blended learning, depending on the school’s curriculum, student ages, and/or selected days

#### Predictions

The mothers believed that education would be enhanced, in the next academic year, if it returned to face-to-face learning or pursued a blended learning approach. They presented several reasons for this belief: First, education would regain its prestige and importance. As one mother said: “I think education will be taken more seriously and its quality will be high when children go to their schools physically.” Second, the level of comprehension of the information and knowledge acquired would increase due to the students attending physical schools and listening to their teachers in person, as well as participating in class activities, communicating with and being monitored by teachers and peers. Third, students would take their deserved marks and be treated fairly according to their efforts. One mother argued: “It will be no more teachers saying I cannot hear you, or I have not received your online homework. These technical issues cut my children’s marks and lowered their academic level.” Fourth, the mothers would have more free time to rest from not having to monitor their children during their studying on the educational platform or having to help their children with their assignments or lessons to enhance their exam performance. As one mother explained: “My children will study forcibly for themselves, because I will not be with them in the classroom, no one will help them to cheat. They must take responsibility for their education.” Another mother commented: “If I rest in the morning, I get enough energy to support my children in their studies after coming home from school.”

Moreover, some mothers added more reasons that they believed enhanced the educational process. These reasons were related to the tremendous technological experience that the educational system gained during the Covid-19 pandemic. The first reason was that children and their families became better adapted to using technology and their digital skills had improved during distance learning. Second, teachers would now apply more digital strategies in their teaching due to the experience they had gained. Third, the use of the internet would increase in schools, in turn supporting the educational process. Fourth, the Ministry of Education would take advantage of this experience and the platform that was built to formally adopt distance learning in critical times, such as during bad weather or another type of emergency situation. Fifth, the value of the computer curriculum and digital skills would grow, and other digital-related courses could be added to the educational system, which would in turn create many job opportunities.

#### Suggestions

Participating mothers made three suggestions for the educational system for the following academic year: provide families with a choice between distance learning and face-to-face learning for their children; divide school time into two periods; and apply blended learning, depending on the school’s curriculum, student ages, and/or selected days. The mothers elaborated on their suggestions in more detail. Some mothers hoped that families would be given the choice between distance or face-to-face learning. Thus, the family would be responsible for choosing the method to be followed.

Some mothers hoped that families would be given the choice between distance or face-to-face learning. Thus, the family would be responsible for its choice.

Other mothers suggested that school time should be divided into two periods: a morning period and an afternoon period. As one mother commented: “This idea will bring more jobs in the educational field, which is very good.” Other mothers discussed this suggestion and improved upon it with more ideas, such as shortening the time spent at school, reducing the number of students in every class to no more than 20 students, and adhering to Covid‐19 precautionary measures.

Some mothers suggested blended learning. As one mother explained: “I suggest that practical curricula are taught physically at school, while other curricula are taught in distance learning.” Another mother had a different perception: “I suggest that oral-based curricula such as the holy Quran, literacy, and languages are taught face to face because they require the study of facial expressions.” Another mother presented a different suggestion for blended learning: that it be blended not based on curricula but instead on the number of days or weeks. She stated: “I suggest that students should study from home as distance learning for two days, then go to school for the other three days. Or it could be some weeks online and others at school.”

Several mothers emphasized that primary students should receive face-to-face learning because this stage is essential in students’ academic lives. As one mother said: “It is necessary for primary students to have face-to-face learning to get maximum understanding and to build knowledge through their communications with their friends and with teachers.” On the other hand, two mothers suggested that primary students should stay and study at home because it is safer for them from viruses and behavioral problems.

## Discussion

This study investigated the perceptions of female breadwinners from low-income families regarding their children’s distance learning and gathered their predictions about and suggestions for the educational system in the subsequent academic year.

Financial issues were mentioned frequently by the mothers. As discussed above, breadwinners normally worry about money, i.e., how it will be earned and spent. Female breadwinners tend to experience additional pressure regarding the possibility of losing their jobs when they are the sole or primary breadwinner for the household (Meisenbach, 2010). Despite the mothers’ happiness about the decrease of school expenses, they expressed concerns about unexpected costs, such as the increase of electricity bills and internet fees. The mothers expressed their delight that the Ministry of Education was saving money during the pandemic. This in turn reflected the mothers’ love for their country and desire for its future prosperity.

The breadwinners’ thoughts about money reflected their predictions and suggestions for the following academic year, such as their happiness about improvements in their children’s digital skills and increased government interest in these skills, which may ultimately lead to greater job opportunities. Improvements in children’s digital skills were also noted in the study by Misirli and Ergulec (2021), in which the observation was made that distance learning during the Covid-19 pandemic had accelerated the adoption of digital technologies.

The findings indicated that distance learning burdened the mothers with an overload of responsibilities. As breadwinners, the mothers suffered from lack of time and struggled in balancing between job demands, housework, and meeting their children’s learning needs. Further, they were forced to cope with new technology and learn how to deal with the online educational environment. Misirli and Ergulec, (2021) stated that parents’ responsibilities have increased as a consequence of their children’s distance learning, especially with regard to technical issues. In addition, some mothers were saddened that they were not knowledgeable about the content of their children’s curricula. The school closure has been accompanied by a new kind of struggling, ﻿by the feeling of being overwhelmed, and by a sense of guilt among parents who feel incapable of supporting their children’s online learning (Garbe et al., 2020). Parents’ feeling of guilt could also be associated with the lack of time needed to provide their children with sufficient attention while they are learning remotely (Gandolfi et al., 2021). Monitoring students during educational processes is typically the responsibility of teachers, but during distance learning, it became the job of parents. It is important for parents to understand the necessity of their roles as monitors and motivators for their children during distance learning, the nature of which differs markedly from that of face-to-face learning (Churiyah & Sakdiyyah, 2020; Misirli & Ergulec, 2021).

Privacy issues were noted in the current study by some of the mothers, who voiced concerns over accidentally being physically exposed during their children’s online classes. This concern is especially connected to cultural background, as these breadwinners are Saudi Muslims who embrace religious traditions and cultural customs that require women to cover their bodies in front of unrelated men. Therefore, their exposure online, even if by accident, may lead to severe problems among their larger family and in society at large (Al Lily et al., 2021).

Parental protection was expected from the mothers in this study, as they were widows, breadwinners, and the sole guardians of their children. The mothers’ concern over protecting their children was clear in their marked appreciation for distance learning, which had allowed them to monitor their children’s academic performance and behavior inside classes. Thus, they could provide their children with the academic and behavioral assistance they needed.

Even though the mothers’ support of cheating was intended to protect their children, as explained above, they declared that this support would badly affect the quality of learning for their children and would degrade their children’s moral values, which the mothers had endeavored to protect.

Distance learning means that children learn from home, which was appreciated by the mothers who wanted to protect their children from Covid-19 and other diseases. The school closure policy and transition to online learning was aimed at preventing the spread of Covid-19, which the parents agreed was essential to protecting the health and safety of everyone (Churiyah & Sakdiyyah, 2020; Garbe et al., 2020). Distance learning also assisted the mothers who wanted to raise their children in the most optimal way, especially protecting them from the impact of bad friends or peers, with whom they would interact at school during face-to-face learning.

In addition, the parental protection was revealed in the mothers’ suggestions for the following academic year. Some mothers suggested that parents should be given the option to choose either online or face-to-face learning. Other mothers suggested dividing the school day into two periods. Yet other mothers recommended that blended learning be applied depending on the school’s curricula, the students’ age, or the selected days. All of these suggestions reflected the mothers’ concerns regarding the spread of Covid-19 and their willingness to protect their children.

The mothers’ desire to protect their children was clear in their concerns regarding the health issues their children experienced during distance learning: for example, the pains their children suffered in their backs or necks, visual impairment, and addiction to digital devices. These findings are aligned with those of Misirli and Ergulec (2021), who found that 76.9% of the participants raised concerns regarding health issues related to the prolonged amount of time their children spent in front of digital screens compared to their lives before the pandemic.

The mothers expressed deep concern regarding their children’s academic outcomes and the quality of the education that their children had received during the crisis. Further, online learning was accompanied by technical issues, problems with students’ motivation to learn, distractions at home, and poor classroom administration by teachers, all of which negatively affected the quality of education. The mothers had worked hard to compensate for the educational deficiencies faced by their children. Such concerns about the quality of education have been raised in several other studies (Abuhammad, 2020; Garbe et al., 2020; Misirli & Ergulec, 2021). However, these concerns were amplified in this study due to the mothers’ worries about the future careers of their children. As breadwinners who are the primary or sole income earner in their household and who had suffered from impoverished lives, these mothers hoped that their children would eventually help them when they obtained good jobs, which required receiving a good education. 

The mothers mentioned the ease and frequency with which cheating occurred during online assignments and exams, which in turn raised ethical issues and threatened to degrade the moral system that the mothers had sought to build in cooperation with the school.

Some of the mothers voiced practical concerns regarding their children who had just started their first school year, which is an essential stage in their academic lives, in distance learning.

These mothers discussed the importance of being physically present and of face-to-face social interactions in school to develop the various skills young children need to begin their school lives. These findings are in line with those of other studies (Bhamani et al., 2020; Gandolfi et al., 2021; Garbe et al., 2020; Misirli & Ergulec, 2021), which found that parents expressed their concerns regarding the socio-emotional development of their children due to the decreased level of peer interactions during distance learning. Further, Misirli and Ergulec, (2021) and Ewing and Cooper (2021) explained that the need for parental assistance was associated with students’ age level. Students in primary and pre-primary education require further parental engagement in their children’s distance learning, while those in secondary or high school require a lower level of parental assistance. In addition, young children need social interactions and physical activities, neither of which can be effectively provided online. The absence of physical and social communication makes it difficult for children to take online learning seriously. They often perceive this type of learning as a vacation from school and hence waste time sleeping or playing (Bhamani et al., 2020).

## Conclusion, limitation, and implications

### Conclusion

The findings related to the first research question were organized under three themes: (1) financial issues, (2) social and cultural issues, and (3) educational issues. The findings related the second research question were classified according to two themes: predictions and suggestions. Mothers presented general and technological reasons for their predictions for enhancing education in the future and suggested three approaches to teaching and learning for the following academic year.

### Limitation

This study has some limitations that can be addressed in future research. First, the sample for the study was limited to only female breadwinners who had the opportunity to enroll in the KFU program. Further studies should be conducted with breadwinners selected from their homes or workplaces. Second, not recording the focus group discussions created some difficulties for the researcher when attempting to document each word. However, mixing and rearranging groups in the third stage assisted with recovering some missing data.

### Implications

This study investigated the perceptions, future predictions, and suggestions of widowed female breadwinners who were the sole income earners in low-income families regarding their children’s online learning during the Covid-19 pandemic. Parents who live in low-income and disadvantaged communities have probably been more affected by the pandemic than others in relation to their children’s online learning.

As Covid-19 continues to persist worldwide, many countries will likely continue to employ online learning for students. Thus, this study may provide useful information to policy makers by expanding their understanding of parents’ perceptions and involvement in their children’s distance learning, as well as in the further development of the online learning system. The findings demonstrate the lack of training that mothers had received to deal with this type of learning. This implies that the Ministry of Education and school administrations should develop training programs delivered specifically to parents to assist them in supporting their children’s distance learning. Mothers’ perceptions of the educational system in the following academic year may provide valuable suggestions to be considered in the development of online learning policies and training, especially for parents who come from low-income families.

Future studies can compare the perceptions of parents from various social class backgrounds or compare the perceptions of female and male breadwinners. This study was exploratory, as its sample size was small. Therefore, future studies can apply other methodologies that include questionnaires administered to larger samples or longitudinal ethnographies that directly access those in low-income families and societies.

## Data Availability

Not applicable
